# Exploring barriers to access and use of limb prostheses and orthoses in sub-Saharan Africa: A systematic review

**DOI:** 10.4102/ajod.v15i0.1852

**Published:** 2026-03-28

**Authors:** Birhanu M. Addis, Demewoz W. Menna, Claire T. Davies

**Affiliations:** 1 Department of Mechanical and Materials Engineering, Faculty of Engineering and Applied Science, Queen’s University, Kingston, Ontario, Canada; 2 Department of Civil Engineering, Faculty of Engineering and Applied Science, Queen’s University, Kingston, Ontario, Canada

**Keywords:** prostheses, orthoses, sub-Saharan Africa, barriers, upper limb, lower limb

## Abstract

**Background:**

Prosthetic and orthotic devices play a significant role in enhancing the quality of life for individuals with disabilities by improving mobility, health, psychological well-being, and socio-economic opportunities. However, access to these assistive devices remains a considerable challenge, particularly in resource-limited settings.

**Objectives:**

The aim of this systematic review was to explore and categorise the existing barriers and challenges that affect access to limb orthoses and prostheses in sub-Saharan African countries.

**Method:**

A systematic search of seven databases was conducted to identify relevant studies using the search terms: ‘prosthesis’ or ‘orthosis’ and sub-Saharan African countries and variations of each term. Quality assessment of each study was completed using the Grading of Recommendations Assessment, Development and Evaluation (GRADE) scale and the Oxford Levels of Evidence.

**Results:**

Twenty-two journal articles were included for review following database search and screening, evaluating a total of 3726 participants. Results were organised into four generalised themes, and each theme was categorised into sub-themes.

**Conclusion:**

The review identifies multifaceted barriers to accessing and utilising limb prostheses and orthoses in sub-Saharan Africa, primarily driven by economic constraints and cultural barriers. The lack of multidisciplinary rehabilitation teams further contributes to fragmented and inefficient service delivery. Orthotic and prosthetic training centers and colleges are underdeveloped, facing problems such as ineffective teaching methods, outdated curricula, inadequate research facilities, and a shortage of experienced professionals.

**Contribution:**

This review synthesises evidence on barriers to prosthetic and orthotic access in sub-Saharan Africa, categorising challenges into thematic domains and highlighting gaps in service delivery, education, and workforce capacity to inform policy, practice, and future research directions.

## Introduction

Limb amputation constitutes a significant global health concern, as it substantially compromises physical functioning and mobility among affected individuals. In 2017, an estimated 57.7 million individuals worldwide were living with limb loss attributable to traumatic causes (McDonald et al. 2021). The implications of traumatic limb amputations are especially profound in low- and middle-income countries (LMICs), where populations are highly susceptible to road-traffic injuries, occupational hazards, conflict-related trauma, war and unmanaged chronic disease, all of which can lead to amputation (McDonald et al. 2021). Globally, it is estimated that only approximately 10% of individuals living with limb amputation who require prosthetic and orthotic devices and services have access to appropriate care; in lower-income settings, this access may be as low as 5% (Abbady et al. 2022; World Health Organization [WHO] 2017). The impact of limb amputations extends beyond physical health, affecting individuals’ psychological well-being and social integration (Horgan & MacLachlan 2004). While this is not unique to LMICs, limited rehabilitation services, economic insecurity, and stigma may intensify these effects in resource-constrained contexts (Sahu et al. 2016; Tsoulou et al. 2019). These combined effects emphasise the need for comprehensive rehabilitation services and access to assistive technologies in low- resource settings.

A prosthesis is an externally applied device used to replace wholly or partly an absent or deficient limb segment. An orthosis is an externally applied device used to support or modify the structural and functional characteristics of the neuromuscular and skeletal systems (International Organization for Standardization [ISO] 2020). In this review, the terms limb prostheses or prosthetic devices and limb orthoses or orthotic devices refer to assistive devices for the upper and/or lower extremities, and do not include non-limb applications such as breast prostheses or spinal orthoses. Limb orthotic and prosthetic devices play a crucial role in assisting people with limb loss or functional limitations to improve their functioning and increase their potential to live healthy, productive, independent, and dignified lives (WHO 2017). Globally, millions of people depend on these assistive devices to overcome physical limitations and engage in everyday activities. However, despite the potential of these assistive devices, accessing and utilising these products remains a significant challenge in many parts of the world, particularly in LMICs such as those in sub-Saharan Africa (SSA) (Marino et al. 2015).

Sub-Saharan Africa is home to a large and growing population of individuals in need of prosthetic and orthotic care, driven by a high incidence of trauma-related injuries, congenital disabilities, and diseases such as diabetes and vascular conditions (Morgado et al. 2022). Despite this demand, the region faces considerable barriers in effectively delivering and utilising prosthetic and orthotic services. Limited healthcare infrastructure, economic constraints, geographic challenges, and barriers originating from sociocultural beliefs and social stigmatisation are among the many factors that hinder individuals from receiving the necessary devices and care (Aoun, Matsuda & Sekiyama 2015; Manxusa & Botha 2025; Marino et al. 2015; Morgado et al. 2022; Visagie et al. 2025). Considering the unmet demands and poor utilisation of limb orthoses and prostheses, it is essential to systematically explore the factors that hinder the delivery and utilisation of prosthetic and orthotic services in SSA. This systematic review aims to comprehensively identify and categorise the barriers and challenges that affect access and use of limb orthoses and prostheses in SSA countries. By doing so, we seek to provide findings into the existing gaps in healthcare provision and inform future strategies for improving access and utilisation of orthoses and prostheses in the region.

## Research methods and design

The Preferred Reporting Items for Systematic Reviews and Meta-Analyses (PRISMA) protocol (Moher et al. 2010) was implemented as a methodological framework to guide the reporting of this systematic review. Based on the PRISMA protocol, the following steps were included in the study: Establishing clear inclusion and exclusion criteria based on the research question, developing a comprehensive search strategy, systematically screening potential studies, extracting relevant data, and finally conducting a thematic analysis.

### Inclusion and exclusion criteria

This review aimed to include all research exploring the access and use of limb orthoses and prostheses within the SSA region. Inclusion criteria: (1) research article published in peer-reviewed journal, (2) written or translated into English language, (3) year of publication: from 2000 up to December 2023, (4) addressed the use or status of limb prosthesis as a primary research topic, (5) explored the barriers and facilitators in accessing and using limb prosthesis, and (6) study must be completed in SSA countries. Exclusion criteria: (1) articles that include countries outside of SSA countries if the data cannot be separated, and (2) systematic reviews, scoping reviews, commentaries, and conference abstracts.

### Information sources and search strategy

Seven electronic databases were systematically searched in December 2023 to identify relevant journal articles for the review. These databases included CINAHL, Engineering Village, IEEE Xplore, Embase, PubMed, Medline, and Web of Science. All databases were accessed through the institutional subscription of Queen’s University library. Moreover, references from prior reviews and selected articles included in the review were examined. A Boolean search strategy was implemented for each database, utilising the Boolean operators ‘OR’ and ‘AND’ to connect the following search terms: ‘prosthesis’ or ‘orthosis’ and ‘sub-Saharan African countries’. The search terms were used to search the title and abstract of all literature within each database:

(Prosthesis OR Prostheses OR Prosthetic* OR Orthosis OR Orthoses OR Orthotic*)

AND

(Low-income OR Developing Countr* OR Resource-limited OR SSA OR Africa OR Nigeria OR Kenya OR Ghana OR Ethiopia OR Tanzania OR Congo OR Uganda OR Senegal OR Mali OR Sudan OR Zimbabwe OR Cameroon OR Libya OR Madagascar OR Angola OR Somalia OR Rwanda OR Namibia OR Mauritius OR Zambia OR Botswana OR Cape Verde OR Mozambique OR Seychelles OR Burkina Faso OR Niger OR Benin OR Malawi OR Liberia OR Togo OR Gambia OR Chad OR Gabon OR Central African Republic OR Eritrea OR Mauritania OR Sierra Leone OR Cote d’Ivoire OR Ivory Coast OR Djibouti OR Eswatini OR Burundi OR South Sudan OR Lesotho)

To ensure a comprehensive search, the terms low-income, resource-limited, and developing country were added to the search strategy to identify studies that might use these terms instead of specific country names within the SSA region.

### Study selection

The study selection process involved four distinct stages. Firstly, a comprehensive search of electronic databases using the aforementioned search terms identified a pool of potential articles. Secondly, duplicate records were removed using EndNote software. Thirdly, titles and abstracts were independently evaluated by two reviewers to assess their eligibility for inclusion. Fourthly, full-text screening was conducted by the same two reviewers to ensure adherence to the inclusion and exclusion criteria. Conflicts in screening decisions were resolved through discussion between reviewers until consensus was reached.

### Data extraction and quality assessment

Data extracted from each article include author, country, study purpose, study design, method of data collection, type of amputation or disability, participants, and outcomes. Quality assessment of each study was completed using the GRADE evaluation scale (Guyatt et al. 2008) and the Oxford Levels of Evidence (OLE) (CEBM 2011).

### Ethical considerations

This article followed all ethical standards for research without direct contact with human or animal subjects.

## Results

### Study identification and selection

**FIGURE 1:**
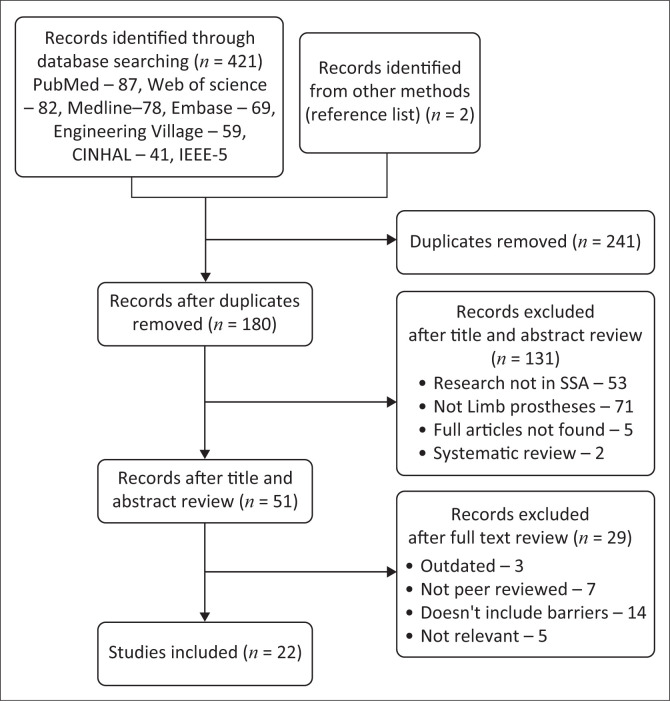
Database searching and screening.

After conducting a comprehensive database search and applying rigorous eligibility criteria, a total of 22 journal articles were selected for full review (Figure 1). Because of the variability of outcomes and the limited availability of quantitative data, a meta-analysis was not feasible.

### Characteristics of studies

The detailed characteristics of each study, including the study design, purpose, data collection method, and type of amputation, are presented in Table 1. Among the studies reviewed, two were case studies (Aduayom-Ahego 2020; Sterkenburg et al. 2021); three employed a mixed-methods design combining qualitative and cross-sectional methods (Aduayom-Ahego, Ehara & Kpandressi 2017; Allen et al. 2022; Andregard & Magnusson 2017). Nine studies employed qualitative methodologies (Dordunu et al. 2024; Ennion & Johannesson 2018; Ennion & Manig 2019; Ennion & Rhoda 2016; Magnusson 2019; Magnusson & Ahlström 2012; Magnusson, Shangali & Ahlstrom 2016; Mattick et al. 2023; Urva et al. 2023); one study adopted a mixed-methods approach with a longitudinal sequential explanatory design (Ennion, Johannesson & Rhoda 2017); another study focused on co-design, development, and testing (Hussaini et al. 2023); four were cross-sectional studies (Magnusson & Ahlström 2017; Magnusson & Bickenbach 2020; Magnusson et al. 2014; Ngarambe et al. 2022); two were cohort studies (Van der Stelt et al. 2021; Von Kaeppler et al. 2021).

**TABLE 1:** Study characteristics.

Record	Purpose	Type of impairment	Data collection method	Sample size	Study design
Record 1 (Andregard & Magnusson 2017)	To describe the experiences of attitudes in the society of Sierra Leone from the perspective of individuals with poliomyelitis and people with amputations using orthotic or prosthetic devices	Lower limb amputation and disability caused by polio	Interviews were conducted using open-ended questions.	12 participants (6-Amputation 6-polio)	A qualitative cross-sectional study
Record 2 (Aduayom-Ahego 2020)	To apply silicone finger prostheses on a patient with multiple finger amputations	Upper limb: multiple-digit amputations in both hands	Patient Engagement or participatory design.	1 participant	Case report
Record 3 (Dordunu et al. 2024)	To explore the experiences of lower limb prosthetic users after prosthetic rehabilitation in a prosthetic and orthotic rehabilitation centre in the Eastern Region of Ghana	Lower limb amputation	A purposive sampling technique and semi-structured interview guide.	17 participants	An exploratory descriptive qualitative design
Record 4 (Ennion & Johannesson 2018)	To explore therapists’ experiences with providing pre-prosthetic rehabilitation in a rural setting	Lower limb amputation	Data were collected from purposively sampled therapists in five district hospitals.	17 participants	A qualitative descriptive approach
Record 5 (Ennion et al. 2017)	To test and explore the clients’ perspectives with the application of prosthetic socket system in a rural community	Lower limb: unilateral trans-tibial amputation	The Orthotic and Prosthetic User’s Survey was administered. Data were collected at 1-, 3- and 6-months post fitting, and two FGDs were also administered.	21 participants	A mixed-methods approach, a longitudinal sequential explanatory design
Record 6 (Ennion & Manig 2019)	To explore the experiences of lower limb prosthetic users and to understand the importance of a lower limb prosthesis to a prosthetic user in a rural area of South Africa	Lower limb amputation	A semi-structured interview guide was used to collect data from prosthetic users in a rural area.	9 participants	A generic qualitative approach and an explorative design
Record 7 (Ennion & Rhoda 2016)	To explore the roles and challenges of the members of the multidisciplinary team involved in trans-tibial amputation rehabilitation in a rural community in South Africa	Lower limb: trans-tibial amputation	Semi-structured interviews and focus group discussions.	40 participants	An explorative sequential qualitative descriptive study
Record 8 (Hussaini et al. 2023)	To address the specific problem of creating a terminal device that is culturally acceptable in Uganda and how 3D printing efficiently facilitates arriving at a solution	Upper Limb	Group discussion to obtain feedback from participants.	21 participants	Co-design, development, and testing
Record 9 (Magnusson 2019)	To compare and synthesise findings related to experiences of prosthetic and orthotic service delivery in Tanzania, Malawi, Sierra Leone and Pakistan from the perspective of local professionals	N/A	Individual interviews conducted. The second-order concept analysis was applied to the data.	49 participants	A qualitative inductive study
Record 10 (Magnusson & Ahlström 2012)	To explore the experiences of prosthetic and orthotic service delivery in Sierra Leone from the local staff’s perspective	N/A	Semi-structured individual interviews were conducted	15 prosthetic and orthotic technicians	An explorative qualitativedesign
Record 11 (Magnusson & Ahlström 2017)	To investigate similarities and differences between Sierra Leone and Malawi concerning participants’ mobility and satisfaction with their lower limb prosthetic or orthotic device and related service delivery, and to identify variables associated with patients’ satisfaction with assistive devices and associated services in the entire study group from these two low-income countries	Lower limb	Questionnaires, including QUEST.	A total of 222 participants – Sierra Leone (139) and Malawi (83)	A cross-sectional survey study in two low-income countries with a correlative and comparative design
Record 12 (Magnusson & Bickenbach 2020)	To evaluate the access to human rights of persons with disabilities who use prosthetic and orthotic assistive devices, and to compare groups of participants in terms of gender, residential area, income, and type and level of assistive device	Lower limb	Questionnaires were used to collect self-reported data	139 participants	A cross-sectional study
Record 13 (Magnusson et al. 2014)	To investigate patients’ mobility and satisfaction with their lower limb prosthetic or orthotic device and related service delivery in Sierra Leone; to compare groups of patients regarding type and level of assistive device, gender, area of residence, income; and to identify factors associated with satisfaction with the assistive device and service	Lower limb	Questionnaires, including the Quebec User Evaluation of Satisfaction with Assistive Technology questionnaire (QUEST 2.0)	139 participants	A cross-sectional study
Record 14 (Magnusson et al. 2016)	To describe how Tanzanian and Malawian graduates of the Diploma in Orthopaedic Technology perceive their education and how it could be improved or supplemented to facilitate the clinical practice of graduates	N/A	Individual semi-structured interviews	19 participants	A qualitative phenomenographic approach
Record 15 (Mattick et al. 2023)	To explore the personal and system factors that motivate and enhance outcomes for patients accessing a prosthetic service and using a lower limb prosthesis within a low-resource setting	Lower limb	Semi-structured interviews	10 participants	A qualitative approach
Record 16 (Ngarambe et al. 2022)	To establish the number of persons with lower limb amputation with or without prosthesis and to determine their socio-economic profile in Rwanda	Lower limb	Telephone interviews were conducted to complete the questionnaire	3026 participants	A descriptive, cross-sectional study
Record 17 (Sterkenburg et al. 2021)	To investigate the impact of a 3D printed prosthesis on the health-related quality of life (HRQoL) in prosthesis recipients	Upper Limb	EQ-5D-5L questionnaire – follow-up interviews	7 participants	Case study
Record 18 (Urva et al. 2023)	To examine the burden of transfemoral amputation and barriers to prosthesis provision as perceived by patient, caregiver and healthcare professional, at a single tertiary referral hospital in Tanzania	Lower limb – Transfemoral amputation	Semi-structured in-depth interviews	5 patients, 4 caregivers, and 11 healthcare providers	Qualitative study
Record 19 (Van der Stelt et al. 2021)	To produce low-cost 3D-printed trans-tibial prosthetic sockets and conduct tests in a rural area of Sierra Leone	Lower limb: trans-tibial amputation	Computer-aided design (CAD) and computer-aided manufacturing (CAM)	8 participants	Co-design, product development and observational cohort study
Record 20 (Von Kaeppler et al. 2021)	To quantify the impact of prostheses on the quality of life and function in Tanzanian transfemoral amputees	Lower limb – Transfemoral amputation	EuroQol-5D-3L (EQ-5D-3L), (PLUS-M), 2-minute walk test (2MWT) and Physiologic Cost Index (PCI).	30 participants	A prospective cohort study
Record 21 (Allen et al. 2022)	To explore the perceived barriers that lower limb amputees and service providers face when accessing or providing rehabilitation services. The secondary aim was to describe the lower limb amputations performed in public hospitals in the Western Area of Sierra Leone.	Lower limb	Data collected from surgical logbooks and interviews with amputees and group discussion and interviews with service providers	Amputees (*n* = 10) Service providers (*n* = 11) Total – 21 participants.	Mixed-methods cross-sectional study
Record 22 (Aduayom-Ahego et al. 2017)	To explore the perceptions of students and alumni regarding the challenges in prosthetics and orthotics programme in a sub-Saharan African francophone country Togo.	N/A	Survey questionnaires	12 alumni and 19 students Total – 31 participants	Cross-sectional and qualitative study

Note: Please see the full reference list of the article, Addis, B.M., Menna, D.W. & Davies, C.T., 2026, ‘Exploring barriers to access and use of limb prostheses and orthoses in sub-Saharan Africa: A systematic review’, *African Journal of Disability* 15(0), a1852. https://doi.org/10.4102/ajod.v15i0.1852, for more information.

N/A, not applicable.

**FIGURE 2:**
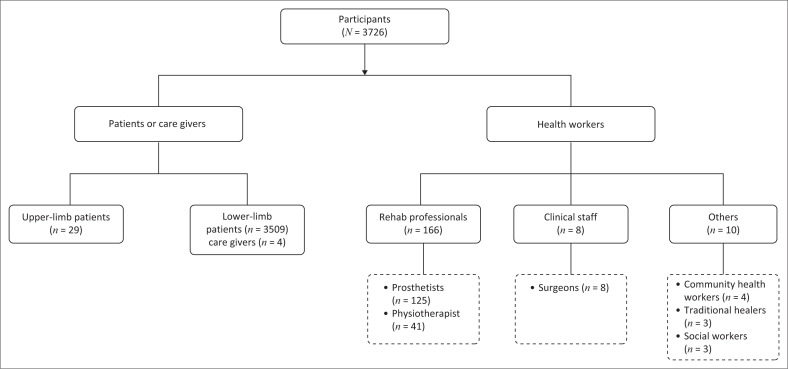
Distribution of participants by group across the selected studies.

The participants in the studies can be broadly categorised into two groups: (1) patients, and (2) health professionals, including orthotists and prosthetists, caregivers, surgeons, physiotherapists, and community health workers, as illustrated in Figure 2.

Of the 22 studies selected for review, Sierra Leon had the highest number of articles on limb prosthesis. South Africa ranked second, with four articles, as shown in Table 2. Two studies were conducted across two countries, and one study covered three countries.

**TABLE 2:** Number of articles per country.

Number	Country	Number of articles
1	Sierra Leone	7
2	South Africa	4
3	Ghana	2
4	Tanzania	2
5	Uganda	1
6	Rwanda	1
7	Kenya	1
8	Togo	1
9	Tanzania and Malawi	1
10	Sierra Leone and Malawi	1
11	Tanzania, Malawi, and Sierra Leone	1

**FIGURE 3:**
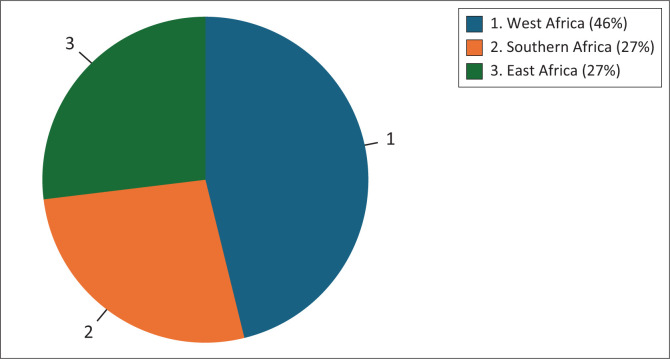
Percentage of articles by region.

Regionally, West Africa contributed nearly half of the included studies (46%), whereas Southern Africa and East Africa each accounted for 27% of the studies, as shown in Figure 3.

### Quality assessment of studies

The quality of the 22 studies included in this systematic review was evaluated using the OLE and GRADE systems as shown in Table 3. First, the study design of each article was classified within the Oxford hierarchy (Levels 1–5) based on methodological strength. Based on OLE, the two cohort studies were assigned Level 2b, reflecting their observational design, while the longitudinal sequential explanatory mixed-methods study was designated Level 3, consistent with its quantitative longitudinal component. However, most other studies such as case studies, co-design and testing, mixed-methods, and cross-sectional were rated Level 4, with qualitative studies receiving the lowest ranking at Level 5 due to their descriptive, non-controlled nature within the OLE hierarchy. Considering that the Oxford hierarchy prioritises study design over methodological rigour and may undervalue well-conducted qualitative or mixed-methods research, its limitations were mitigated by complementing the OLE classification with a more detailed appraisal of methodological quality using the GRADE system.

**TABLE 3:** Oxford levels of evidence and GRADE assessment.

Study design	Number of studies	Oxford level	Initial GRADE	Final GRADE
Case studies	2 Records (Aduayom-Ahego 2020; Sterkenburg et al. 2021)	4	Low	Low
Mixed-methods (Qualitative + Cross-sectional)	3 Records (Aduayom-Ahego et al. 2017; Allen et al. 2022; Andregard & Magnusson 2017)	4	Low	Low
Qualitative methodologies	9 Records (Dordunu et al. 2024; Ennion & Johannesson 2018; Ennion & Manig 2019; Ennion & Rhoda 2016; Magnusson 2019; Magnusson & Ahlström 2012; Magnusson et al. 2016; Mattick et al. 2023; Urva et al. 2023)	5	N/A	N/A
Mixed-methods (Longitudinal sequential explanatory)	1 Record (Ennion et al. 2017)	3	Low	Low
Co-design, development, testing	1 Record (Hussaini et al. 2023)	4	Low	Low
Cross-sectional studies	4 Records (Magnusson & Ahlström 2017; Magnusson & Bickenbach 2020; Magnusson et al. 2014; Ngarambe et al. 2022)	4	Low	*Moderate – 3* (Magnusson & Ahlström 2017; Magnusson et al. 2014; Ngarambe et al. 2022) *Low* – 1 (Magnusson & Bickenbach 2020)
Cohort studies	2 Records (Van der Stelt et al. 2021; Von Kaeppler et al. 2021)	2b	Low	Low

Note: Please see the full reference list of the article, Addis, B.M., Menna, D.W. & Davies, C.T., 2026, ‘Exploring barriers to access and use of limb prostheses and orthoses in sub-Saharan Africa: A systematic review’, *African Journal of Disability* 15(0), a1852. https://doi.org/10.4102/ajod.v15i0.1852, for more information.

GRADE, Grading of Recommendations Assessment, Development and Evaluation; N/A, not applicable.

Under the GRADE system, all quantitative studies (*n* = 13), excluding qualitative studies, were initially assigned a low-quality rating because of their observational designs, with potential adjustments considered for risk of bias to determine final ratings (ranging from High to Very Low). Following an evaluation of risk of bias, three cross-sectional studies (Magnusson & Ahlström 2017; Magnusson et al. 2014; Ngarambe et al. 2022) were upgraded to a Moderate GRADE rating, as they included a substantial number of participants and employed standardised survey questionnaires, enhancing the reliability and precision of their findings.

### Thematic analysis: Barriers to access and use of limb orthoses and prostheses

The primary barriers and challenges to accessing and using limb orthoses and prostheses in the SSA region are identified and discussed from the search results and comprehensive analysis of the materials gathered during the review process. These barriers are organised into four generalised themes, and each theme is categorised into sub-themes.

#### Theme 1: Barriers to access limb orthoses and prostheses

**Socio-economic barriers:**

- Poverty: Studies (Andregard & Magnusson 2017; Ennion & Johannesson 2018; Ennion & Manig 2019; Magnusson et al. 2014; Ngarambe et al. 2022) reported that patients with limb loss struggle with poverty as they have limited access to employment, and this affects the possibility of accessing rehabilitation services.
- Limited financial support: Studies reported that government financial support systems for people with disabilities are often inadequate or nonexistent, leaving individuals to cover the cost of prosthetic service (Andregard & Magnusson 2017; Ennion & Johannesson 2018; Magnusson 2019; Magnusson & Ahlström 2012).

**A lack of resources and poor infrastructure of healthcare services:**

- Challenges with access to prosthetic service centres: Access to prosthetic services is often limited because of the scarcity of specialised clinics and rehabilitation centres. Key barriers include long distances to service centres, limited availability of repair services, high rehabilitation costs associated with the private sector, long waiting times for services in governmental sectors, and significant travel expenses, especially in rural areas, all of which restrict access for those in need (Allen et al. 2022; Ennion & Rhoda 2016; Magnusson & Bickenbach 2020).
- A lack of prosthetic supplies and materials: Five records (Ennion & Johannesson 2018; Ennion & Manig 2019; Ennion & Rhoda 2016; Magnusson 2019; Magnusson & Ahlström 2012) emphasised that insufficient government support has led to a shortage of materials and equipment in rehabilitation centres, limiting their capacity to produce new prostheses or to maintain existing ones.
- Staff shortages: Studies (Ennion & Johannesson 2018; Ennion & Rhoda 2016) highlighted that there is a shortage of trained prosthetists and orthotists who can fit and maintain prosthetic limbs, as well as provide necessary rehabilitation services.

**Cultural barriers:**

- Stigma: In some of the studies (Andregard & Magnusson 2017; Dordunu et al. 2024; Magnusson & Ahlström 2012), it is reported that the stigma associated with disability and the use of prosthetics leads to social exclusion or discrimination.
- Traditional beliefs: Studies (Andregard & Magnusson 2017; Ennion & Johannesson 2018; Ennion & Rhoda 2016; Magnusson & Ahlström 2012) indicate that patients in rural areas often perceive disabilities as a result of bewitchment or curses, leading them to seek assistance from traditional healers. This belief fosters feelings of discouragement, low self-esteem, and social isolation, which, in turn, reduces their motivation to explore biomedical rehabilitation options, such as prosthetic services.
- Choice of service provider: In some cases, culture also affected the patients’ choice of service provider, in which patients refused to be treated by therapists from their own area and race (Ennion & Johannesson 2018).

**Limited awareness and prioritising of prosthetic and orthotic services:** Some studies (Ennion & Rhoda 2016; Magnusson & Ahlström 2012) reported that there is limited public awareness about the availability and benefits of prosthetic and orthotic services. This lack of knowledge prevented patients from seeking prosthetic care or knowing what options are available. Health workers have observed that family members may urge patients to consult traditional healers before pursuing biomedical options.

#### Theme 2: Challenges in the use of limb orthoses and prostheses

**Discomfort from prosthesis:** Several studies have reported discomfort associated with the use of prosthetic limbs, both upper and lower. Seven records specifically discussed challenges related to lower limb prosthesis use (Allen et al. 2022; Dordunu et al. 2024; Ennion et al. 2017; Magnusson & Ahlström 2017; Magnusson et al. 2014; Mattick et al. 2023; Von Kaeppler et al. 2021). Common problems identified include excessive sweating and itchiness, as noted in studies (Dordunu et al. 2024; Ennion et al. 2017; Magnusson & Ahlström 2017) while pain was a significant concern in studies performed by Mattick et al. (2023), Dordunu et al. (2024), Magnusson et al. (2014) and Magnusson and Ahlström (2017). Moreover, Mattick et al. (2023) and Ennion et al. (2017) discussed the development of wounds in the residual limb as a further complication. In terms of upper limb prostheses, one study identified skin-related issues as a primary source of discomfort (Sterkenburg et al. 2021).

**Size and fitting problems:** Size and fitting problems were noted in both lower and upper limb prostheses across different studies. One study (Magnusson & Ahlström 2017) reported inadequate sizing as a key issue in lower limb prostheses, while two studies (Sterkenburg et al. 2021; Von Kaeppler et al. 2021) identified difficulties with the fitting of prostheses further diminish user satisfaction that leads to abandonment.

**Challenges while walking on different surfaces:** Four studies (Dordunu et al. 2024; Ennion et al. 2017; Magnusson & Ahlström 2012; Von Kaeppler et al. 2021) focusing on lower limb prostheses have documented difficulties in using lower limb prostheses while walking on uneven surfaces, muddy terrain, and navigating hills. These findings underscore the functional challenges associated with prosthetic use in environments that are not level or stable.

**Absence of the desired functionality:** Three studies on upper limb prostheses (Aduayom-Ahego 2020; Hussaini et al. 2023; Sterkenburg et al. 2021) discussed limitations in functionality, particularly concerning restricted hand movement and finger dexterity. Moreover, a participant using a lower limb prosthesis (Allen et al. 2022) reported that the device significantly slowed her down, ultimately leading to its abandonment.

**Prosthesis longevity problem:** Two studies (Allen et al. 2022; Von Kaeppler et al. 2021) highlight that prosthesis failure, combined with inadequate maintenance, significantly reduces the lifespan of the devices.

**Limited physiotherapy options:** The rehabilitation progress is limited as users with newly fitted prostheses receive limited access to physiotherapy services (Von Kaeppler et al. 2021).

**Experiencing a loss of self-confidence when utilising the prosthesis:** Patients discontinued wearing their prostheses between follow-up appointments, expressing that they felt more self-confident without the prosthesis than when they had been using it (Sterkenburg et al. 2021).

#### Theme 3: Challenges from rehabilitation professionals’ perspective

The review identified six studies (Allen et al. 2022; Ennion & Johannesson 2018; Ennion & Rhoda 2016; Magnusson 2019; Magnusson & Ahlström 2012; Urva et al. 2023) that examined barriers from the perspective of rehabilitation professionals. Among the studies, three studies exclusively involved rehabilitation professionals as participants, while the remaining three included a mixed cohort of both patients and rehabilitation professionals. The most common barriers identified by rehabilitation professionals across the six studies are also discussed in theme 1, which are as follows: a lack of resources and poor infrastructure of healthcare services; staff shortages; a lack of government support; cultural factors and awareness problems. The following challenges and barriers are additional barriers from professionals’ perspective specific to each study.

**Lack of multidisciplinary rehabilitation team (MDT):** One study (Ennion & Rhoda 2016) reported that patients, prosthetists, therapists, surgeons, and traditional healers all emphasised the absence of MDT rehabilitation. The lack of coordination among team members adversely affected the rehabilitation process. Similarly, another study (Magnusson 2019) found that operative planning for amputations was predominantly determined by surgeons, often without input from prosthetists, which could limit the suitability of the stump site for prosthetic fitting.

**Lack of referral and follow-up:** The success of any rehabilitation programme relies heavily on patient adherence and consistent follow-up by physiotherapists. In this context, one study (Ennion & Johannesson 2018) reported significant challenges in patient follow-up for rehabilitation and treatment, primarily because of the patients’ poor socio-economic status.

**Working in underserviced and less-resourced settings:** A study (Magnusson 2019) reported that rehabilitation professionals struggled in rural workshops to provide services to long-neglected patients with complex needs.

**Inadequate counselling:** A mismatch exists between patient expectations and actual outcomes because of inadequate counselling on prosthetic use, which ultimately leads to abandonment of the prosthesis (Magnusson 2019).

**Language barrier:** One study (Ennion & Rhoda 2016) reported that rehab professionals faced language barriers, which adversely impacted their ability to effectively diagnose, counsel, and deliver high-quality rehabilitation services to patients.

#### Theme 4: Barriers surrounding prosthetic and orthotic education

Two studies (Aduayom-Ahego et al. 2017; Magnusson et al. 2016) focused on prosthetic and orthotic education in the SSA region: The first study (Aduayom-Ahego et al. 2017) included both graduates and current students, whereas the other study (Magnusson et al. 2016) focused only on graduates. The studies discussed the challenges and barriers in prosthetic and orthotic education as follows:

**Low awareness of the profession:** In a study (Magnusson et al. 2016), participants reported having limited knowledge about the prosthetics and orthotics profession before beginning their education. Because of minimal promotion of the field, it only attracts few potential students.

**A lack of resources:** A Record (Aduayom-Ahego et al. 2017) indicated a shortage of research equipment and facilities, specifically a lack of biomechanics tools and motion analysis systems. Similarly, another study (Magnusson et al. 2016) highlighted a scarcity of textbooks focused on prosthetic and orthotic technology.

**Ineffective teaching methods and outdated curriculum:** According to a Record (Magnusson et al. 2016), teaching methods in the field emphasise theoretical content over clinical training and are not oriented towards problem-solving skills. Moreover, the study found that the curriculum doesn’t include advanced prosthetic and orthotic technology.

**Shortage of experienced professionals:** Both studies (Aduayom-Ahego et al. 2017; Magnusson et al. 2016) identified a shortage of experienced faculty members in the field.

**Limited research funding:** A Record (Aduayom-Ahego et al. 2017) found an insufficiency of research funding for prosthetic and orthotic studies.

**A lack of continued education:** Both studies (Aduayom-Ahego et al. 2017; Magnusson et al. 2016) pointed to a lack of available graduate programmes, limiting opportunities for continued education in the field.

**Inequality in treatment of students:** Local students receive preferential treatment compared to international students (Magnusson et al. 2016).

## Discussion

This systematic review aims to comprehensively identify and categorise the barriers and challenges that affect access to limb orthoses and prostheses in SSA countries. The review identified four main themes with each categorised into sub-themes. The four main themes are: (1) Barriers to access limb orthoses and prostheses, (2) Challenges while using limb orthoses and prostheses, (3) Challenges from rehab professionals’ perspective, and (4) Challenges surrounding prosthetic and orthotic education.

Based on the International Classification of Functioning, Disability, and Health (ICF), under the contextual factors, the experiences of prosthesis and orthosis users are influenced by a combination of environmental and personal factors (Jarl & Ramstrand 2018). Within the ICF framework, challenges and barriers identified under the first and second theme can be categorised into these two domains. Environmental factors include discomfort caused by prostheses, issues with size and fitting, difficulties in navigating different surfaces while walking, a lack of desired functionality, limited access to physiotherapy, poverty, society’s traditional beliefs, a lack of support from government, long distances to service centres, insufficient repair services, and the high costs associated with travel and rehabilitation. On the other hand, the personal factors are a loss of self-confidence when using prostheses, reduced motivation because of stigma, language barriers, personal beliefs, and choice of service provider. External factors significantly influence the experiences of prosthesis and orthosis users. It is crucial for professionals involved in the design and provision of these devices to consider these factors carefully to enhance user satisfaction and functionality. At the governmental level, policies should address the challenges faced by rural prosthesis users, ensuring that they are better integrated into the rehabilitation sector through improved accessibility and targeted support initiatives.

The review discussed several barriers from the rehabilitation professionals’ perspective in providing care to persons with amputations. Many of these barriers align with those identified by people with disabilities, the primary participants in the rehabilitation process. Also, the review revealed two unique and significant challenges highlighted by rehabilitation professionals. Firstly, the lack of an MDT approach in rehabilitation was identified as a critical barrier. Multidisciplinary rehabilitation is widely recognised as the gold standard in prosthetic care in developed countries, as it integrates the expertise of diverse healthcare professionals, including patients, prosthetists, physiotherapists, surgeons, and social workers. This approach addresses the holistic needs of individuals requiring physical rehabilitation services (Esquenazi 2004). However, the review found that MDT rehabilitation is often unavailable in resource-limited settings, particularly in rural areas because of factors such as insufficient resources, a shortage of trained professionals, and the absence of frameworks for MDT implementation (Ennion & Rhoda 2016; Magnusson 2019). The lack of coordination among team members was shown to negatively impact the rehabilitation process, patient outcomes, and overall quality of life. Secondly, rehabilitation professionals reported working in severely understaffed and under-resourced environments (Ennion & Johannesson 2018; Ennion & Rhoda 2016; Magnusson 2019). These challenging conditions were found to reduce productivity and limit the effectiveness of rehabilitation services, further compounding the barriers faced by individuals with amputations.

The review indicated that there is a notable disparity in research focus between upper and lower limb prostheses, with studies on upper limb prostheses accounting for only 13% of the total research on limb prostheses. This difference might be because of the higher demand for lower limb prostheses, as lower limb function is essential for mobility and ambulation, which directly affects the quality of life and independence of individuals with limb amputation. Moreover, the design and functionality requirements for upper limb prostheses present unique challenges. Unlike lower limb devices, upper limb prostheses need to replicate complex movements and dexterous capabilities of the hand, fingers, and arm, requiring greater flexibility, multijoint articulation, and fine motor control. These technical challenges contribute to the limited provision and research on upper limb prosthetics, as achieving functional performance comparable to a natural limb requires advanced engineering and intricate design. As a result, research prioritises lower limb prostheses because of the comparatively straightforward design requirements and broader applicability in addressing mobility needs.

This review has also observed that the included studies predominantly focused on barriers related to limb prosthetic services, products, and the experiences of prosthesis users, with comparatively limited attention given to orthotic services and experiences of orthotic users. The few studies that address orthotic services do so within a broader scope by either focusing on prosthetic and orthotic education or presenting the perspectives of rehabilitation professionals on both prosthetic and orthotic services. However, this imbalance in the review should not be interpreted as reflecting a lower need for orthotic services. Evidence suggests that, in some contexts, a greater number of individuals seek orthotic treatment than prosthetic care (ICRC 2013). One possible explanation for the under-representation of orthosis-focused studies in this review relates to the search strategy employed. Specifically, the search terms did not explicitly include device-specific orthotic terminology (e.g. ankle-foot orthoses, thoracolumbar sacral orthoses, insoles, calipers etc.). This omission likely contributed to the exclusion of certain relevant studies such as (Kelemework et al. 2016; Rios et al. 2022; Visagie, Hunt & Deist 2026).

The systematic review also included the challenges in Prosthetic and Orthotics (P&O) education in the region as the availability of well-trained orthotists and prosthetists directly influences the accessibility and utilisation of limb prostheses and orthoses. Currently, there are only four P&O training programmes in SSA region recognised by the International Society of Prosthetics and Orthotics (ISPO): (1) University of Rwanda, (2) Tshwane University of Technology in South Africa, (3) Tanzania Training Centre for Orthopaedic Technologists (TATCOT) in Tanzania, and (4) Ecole Nationale des Auxiliaires Médicaux (ENAM) in Togo. The limited number of ISPO-recognised programmes contributes to a regional shortage of qualified prosthetists and orthotists (International Society for Prosthetics and Orthotics 2024). In addition to the ISPO-accredited institutions, several countries in SSA operate locally established P&O training programmes. Examples include the Br. Tarcisius Prosthetics and Orthotics Training College (BTPOTC) in Ghana, the Institut Supérieur d’Études Paramédicales (ISEM) in Senegal, and the Federal College of Orthopaedic Technology (FECOT) in Nigeria. These programmes vary in curriculum structure, duration, and level of qualification, and may lack formal international accreditation or standardised competency frameworks. The absence of ISPO recognition does not necessarily imply inadequate training; however, it may reflect variability in educational standards, resource availability, and external quality assurance mechanisms.

The review indicates that P&O training centres across SSA face substantial structural and resource-related constraints, including limited teaching materials, inadequate infrastructure, shortages of experienced faculty, and insufficient funding for research and clinical training. These challenges restrict training capacity and contribute to a workforce output that remains far below internationally recommended standards. Compounding this shortage, evidence suggests that even the limited number of graduates produced are not consistently absorbed into the public health system, as many SSA countries fail to establish sufficiently funded posts to employ them (Mduzana et al. 2020). As a result, a considerable proportion of trained P&O professionals migrate to the private sector or seek alternative employment pathways, despite the persistently high unmet need for prosthetic and orthotic services within public healthcare systems. Collectively, these interconnected training and employment constraints exacerbate workforce shortages and deepen inequities in access to P&O services across the region.

### Limitations

Despite employing a comprehensive search strategy across seven distinct databases, this review may not have included all relevant studies. Potential limitations include publication bias, the exclusion of unpublished studies, constraints within the search strategy (such as the omission of device-specific orthotic terminology), or errors during the screening process. Moreover, this review focused only on peer-reviewed articles in an attempt to preserve methodological quality. Another limitation of this systematic review is the absence of the Grading of Recommendations Assessment, Development and Evaluation Confidence in the Evidence from Review of Qualitative Research (GRADE-CERQual) framework (Lewin et al. 2018) for assessing qualitative studies, which could have provided a structured evaluation of qualitative evidence and strengthened confidence in the findings.

## Conclusion

This systematic review underscores the multifaceted barriers to accessing and using limb prostheses and orthoses in SSA, with the root cause being the socio-economic limitations that contribute to poor healthcare infrastructure, a shortage of trained health professionals, limited financial support for people with disabilities, and a shortage of prosthetists and training centres. Even when prosthetic and orthotic devices are accessed, findings from most included studies indicate that users report some level of dissatisfaction, commonly related to limited functionality, discomfort, pain, fitting issues, and concerns about device longevity and durability. Moreover, the absence of an MDT approach in rehabilitation results in fragmented and inefficient service delivery. In order to address these challenges, a concerted effort is needed from both governmental and non-governmental organisations to formulate a comprehensive strategy. This strategy should focus on enhancing healthcare infrastructure, increasing financial resources and training support, adopting an MDT approach, and developing user-centred prosthetic and orthotic devices. Such measures aim to improve accessibility, functionality, and overall user satisfaction.
